# Sensitization Potential of Dental Resins: 2-Hydroxyethyl Methacrylate and Its Water-Soluble Oligomers Have Immunostimulatory Effects

**DOI:** 10.1371/journal.pone.0082540

**Published:** 2013-11-29

**Authors:** Izumi Fukumoto, Atsushi Tamura, Mitsuaki Matsumura, Hiroyuki Miura, Nobuhiko Yui

**Affiliations:** 1 Fixed Prosthodontics, Department of Restorative Sciences, Division of Oral Health Sciences, Graduate School of Medical and Dental Sciences, Tokyo Medical and Dental University, Tokyo, Japan; 2 Department of Organic Biomaterials, Institute of Biomaterials and Bioengineering, Tokyo Medical and Dental University, Tokyo, Japan; University of South Carolina, United States of America

## Abstract

The immunostimulatory effects of the representative dental resin monomer 2-hydroxyethyl methacrylate (HEMA), a HEMA derivative that does not contain a double bond (2-hydroxyethyl isobutyrate, HEIB), and polymerized water-soluble oligomers of HEMA (PHEMA) were investigated. It is known that expression levels of either or both of CD54 and CD86 in THP-1 cells are increased by exposure to sensitizing substances. In this study, the expression levels of CD54 and CD86, the production of reactive oxygen species (ROS), and the viability of the cells were measured after 24 h of incubation with these materials at different concentrations. The concentrations of the materials that induced the expression of both CD54 and CD86 were low in the following order: NiSO_4_, HEMA, and methyl methacrylate (MMA). These results indicate that these dental resin monomers have lower sensitizing potentials than NiSO_4_. Although HEIB, which lacks a double bond, resulted in negligible ROS production and reduced cytotoxicity than HEMA, it induced the expression of CD54 and CD86. Comparison of the results for HEMA and HEIB indicates that dental resin monomer-induced sensitization may be related not only to the oxidative stress related to the methacryloyl group but also to the structures of these compounds. Of particular interest is the result that a water-soluble PHEMA oligomer with a relatively high-molecular weight also exhibited negligible cytotoxicity, whereas the expression level of CD54 increased after exposure to PHEMA at a high concentration. This result serves as a warning that polymerized substances also have the potential to induce sensitization. This study provides insight into the nature of allergic responses to dental resin materials in clinical use and may facilitate the development of more biocompatible restorative materials in the future.

## Introduction

There has been remarkable progress in the development of dental resin materials in terms of their excellent mechanical properties and bond strength to dentin and enamel [1]. Currently, dental resins are widely used as restorative materials in the clinic to treat caries and as part of fixed and removable prostheses. Resin-based dental materials have advantageous properties in comparison with metal-based materials, including greater biocompatibility and better aesthetic appearance. Additionally, resin-based materials are believed to induce milder allergic responses than metal-based materials. However, allergic reactions to resin-based materials have been reported in the clinic [2,3]. For example, Vamnes demonstrated that among 296 patients, 28% and 8% of them were positive for reactions to nickel ions and resin-based materials, respectively, in a patch test [4]. Because most resin-based materials are polymerized in the oral cavity, the resin monomers are required to have low cytotoxicity and low sensitization potential. Numerous studies have been conducted to assess the cytotoxicity of resin constituents such as 2-hydroxyethyl methacrylate (HEMA) and methyl methacrylate (MMA) [5,6]. Additionally, it has been reported that HEMA causes an irritation reaction and contact dermatitis in guinea pigs [7]. Also, many clinical reports describe that the resin-based materials cause sensitization and allergies [2,4]. Although sensitization to resin-based materials is expected to occur due to unreacted residual monomers [8,9], the sensitization potentials of both monomers and polymers must be characterized because the polymerized resins are present in the oral cavity for several years. Thus, the following questions have arisen: (1) does sensitization to resin-based materials depend on the chemical structure of the monomers? ; (2) is sensitization to resin-based materials related to polymerizable groups (e.g., methacryloyl and acryloyl groups)? ; and (3) is sensitization to resin-based materials dominated by monomers rather than polymers? Determining the answers to these questions is urgent for not only the treatment of patients suffering from allergic responses to dental resins but also for the design new dental resins that can be used in allergy-sensitive patients. 

Sensitization is known to involve multiple stages. When a sensitizing substance comes in contact with the skin, that substance activates dendritic cells in the epidermis such as Langerhans cells. These cells migrate to the regional lymph nodes and present the antigen to lymphocytes. Subsequently, antigen-specific T cells proliferate, and eventually the sensitization state is established. The antigen presentation process requires the expression of major histocompatibility complex (MHC) class II molecules and costimulatory factors including CD40, CD54, CD80, and CD86 [10–12]. On the basis of this molecular mechanism, various researchers have individually reported *in vitro* methods for evaluating the sensitizing potential of materials [13–19]. For example, Python reported that the expression level of CD86 in U937 cells, which are human myeloid cells, is increased by exposure to sensitizing substances [13]. Additionally, Ashikaga reported that the expression levels of CD54 and CD86 in THP-1 cells are increased by exposure to sensitizing substances; this assay is referred to as the human cell line activation test (h-CLAT) [16,17]. Recently, Nukuda suggested that the practical utility of a battery system including the combination of the h-CLAT and direct peptide reactivity assay (DPRA), and the combination of these assays provides a more reliable result for determining the skin sensitization potential of chemical substrates [20]. Notably, the correlation between the results of the h-CLAT and those of the conventional local lymph node assay (LLNA) was found to be 84%, indicating the high reliability of this in vitro assay [21]. 

**Figure 1:**
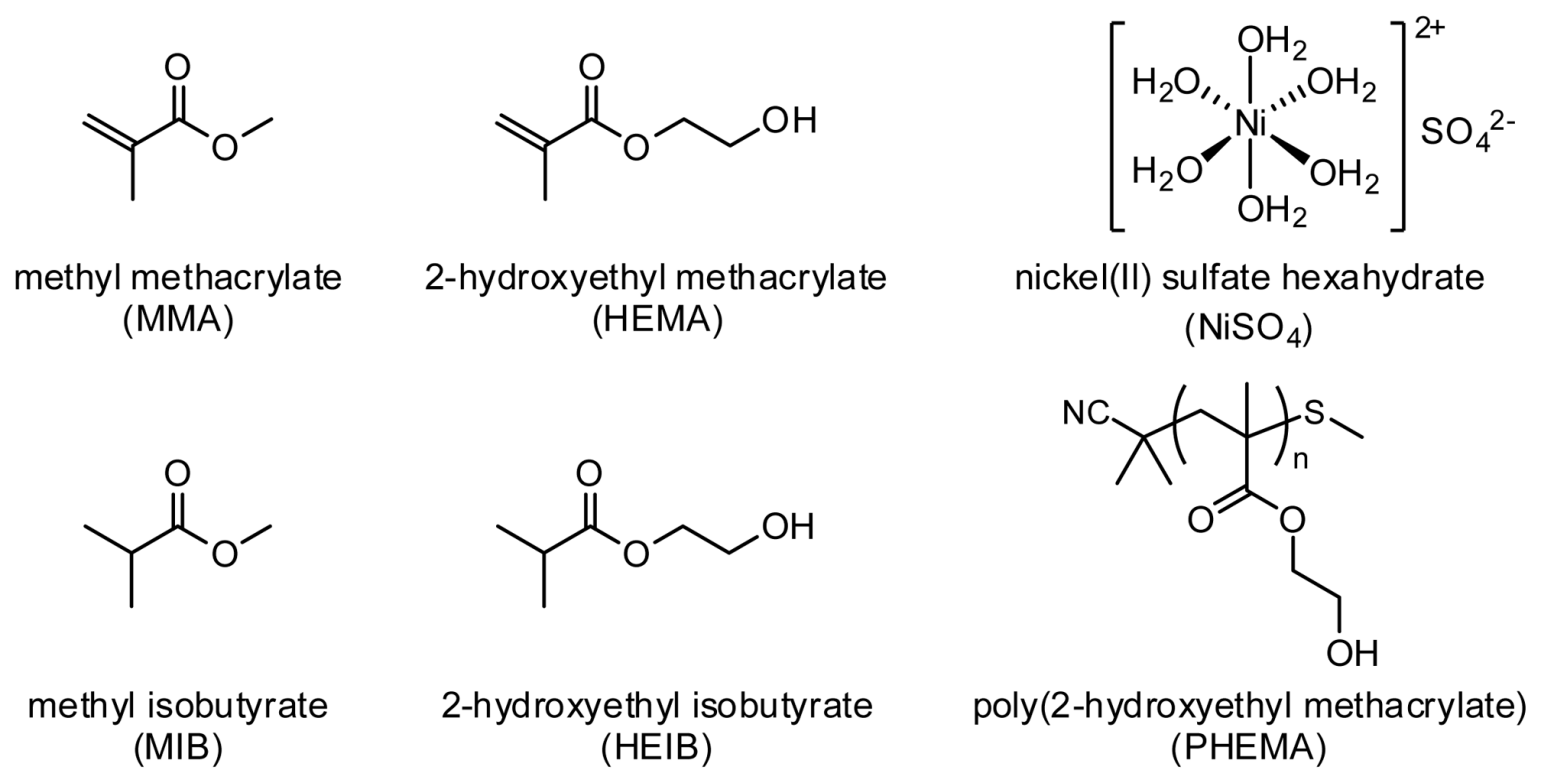
Chemical structures of the samples tested in this study.

In this study, the effect of HEMA, MMA, and nickel ion (a well-known sensitizing agent) on the expression of the costimulatory factors in THP-1 cells, including CD54 and CD86, to estimate their sensitization potential (Figure 1). Next, to better understand how minor change of chemical structure influenced on the cell viability and the expression levels of CD54 and CD86, the effects of the methacryloyl groups of HEMA were investigated. Furthermore, to verify the sensitization potential of dental resin polymers, the effects of water-soluble PHEMA oligomers on the expression levels of CD54 and CD86 in THP-1 cells were investigated. 

## Results

### Expression of CD54 and CD86 in response to HEMA and MMA

**Figure 2:**
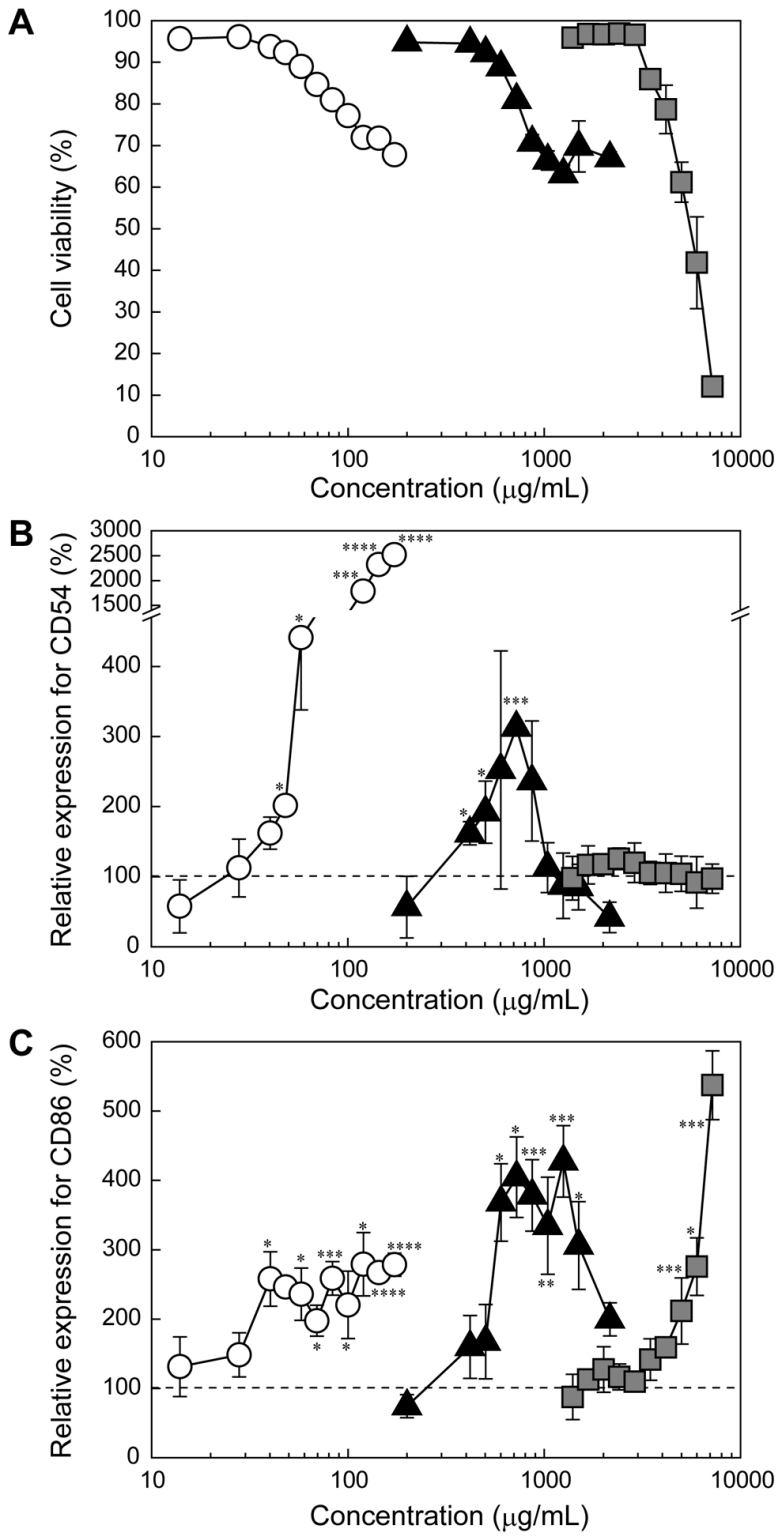
Expression of CD54 and CD86 in THP-1 cells by the treatment with HEMA, MMA and NiSO_4_. Relative viability of (A) and expression levels of CD54 (B) and CD86 (C) in THP-1 cells treated with NiSO_4_ (open circles), HEMA (closed triangles), or MMA (closed squares) at various concentrations for 24 h. The data are expressed as the means ± S.D. (n=3) (*p < 0.05, **p < 0.01, ***p < 0.005, ****p < 0.001).

To evaluate the sensitizing potential of HEMA in cultured cells, THP-1 cells were treated with HEMA for 24 h at various concentrations, and the cell viability and expression levels of CD54 and CD86 were determined by flow cytometry. These results were compared with those for MMA, which has a lower cytotoxicity than HEMA [22]. Additionally, NiSO_4_ was tested as a positive control due to its strong sensitization effect [23,24]. When THP-1 cells were incubated with the test materials, the viability of the cells gradually decreased with increasing concentration for all the test materials (Figure 2). The cell viability curves indicated that the cytotoxicity was low, in the order of MMA, HEMA, and NiSO_4_. Additionally, the expression levels of both CD54 and CD86 increased in a dose-dependent manner after exposure to NiSO_4_, accompanying the reduction in cell viability (Figure 2B, 2C). Notably, NiSO_4_ induced a remarkably high expression level of CD54. HEMA induced significant expression of CD54 at concentrations greater than 400 μg/mL (3.2 mM). At concentrations greater than 723 μg/mL (5.5 mM), the expression level of CD54 decreased, and this decrease was accompanied by a reduction in cell viability. Additionally, HEMA induced the significant expression of CD86 at concentrations greater than 602 μg/mL (4.6 mM). The expression level of CD86 also decreased at concentrations greater than 1,250 μg/mL (9.6 mM) (Figure 2C). MMA had a negligible effect on the expression of CD54 at all tested concentrations, whereas the expression level of CD86 was increased at concentrations greater than 4,166 μg/mL (41.6 mM). Accordingly, the concentrations of materials required to induce the expression of both CD54 and CD86 were low and in the following order: NiSO_4_, HEMA, and MMA.

### Effect of the methacryloyl group of the monomers on the expression levels of CD54 and CD86 and ROS generation

**Figure 3:**
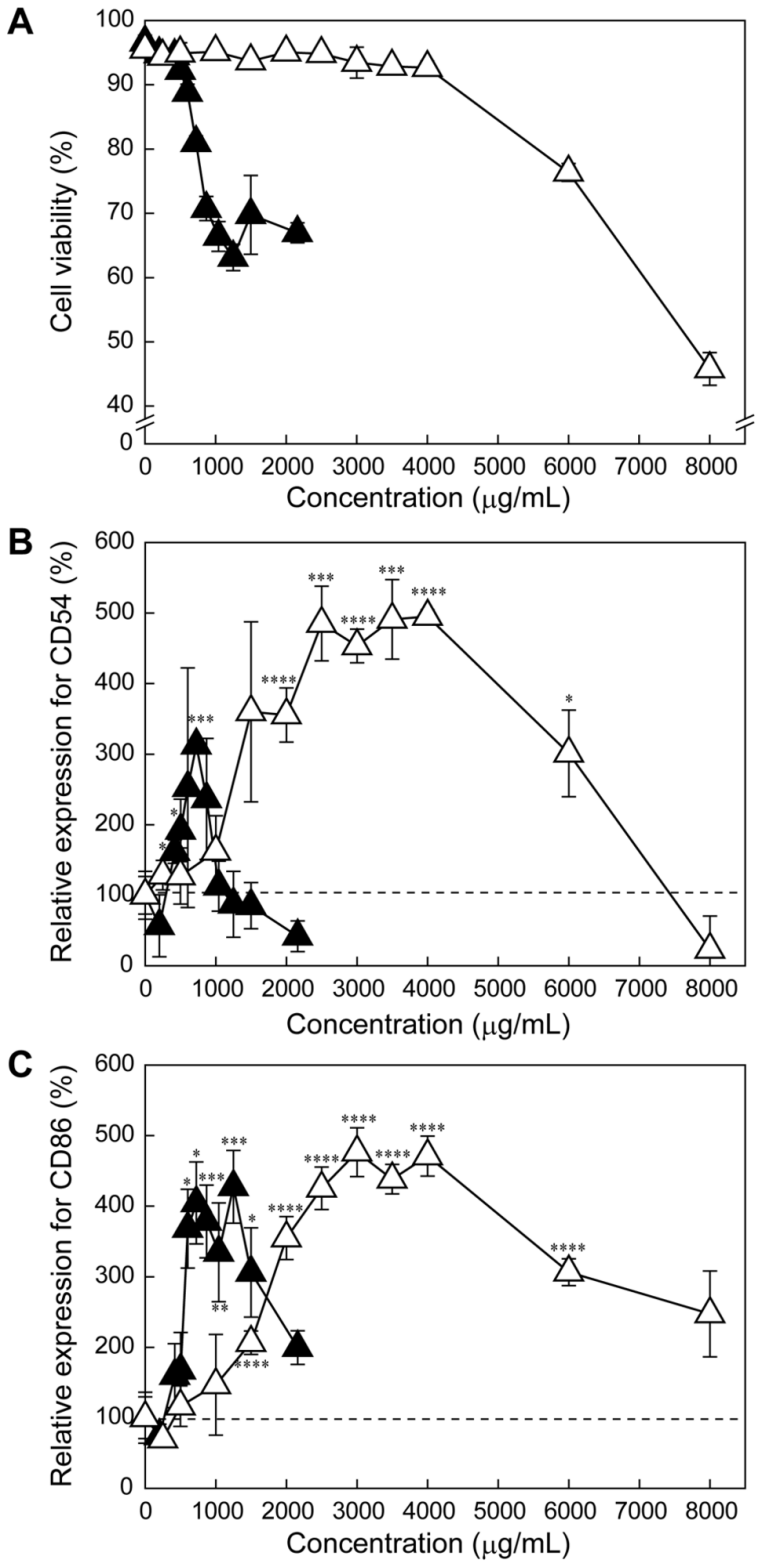
The effect of methacryloyl group of HEMA on the expression of CD54 and CD86. Relative viability of (A) and expression levels of CD54 (B) and CD86 (C) in THP-1 cells treated with HEMA (closed triangles) or HEIB (open triangles) at various concentrations for 24 h. The plots for HEMA were taken from Figure 2. The data are expressed as the means ± S.D. (n=3) (*p < 0.05, **p < 0.01, ***p < 0.005, ****p < 0.001).

**Figure 4:**
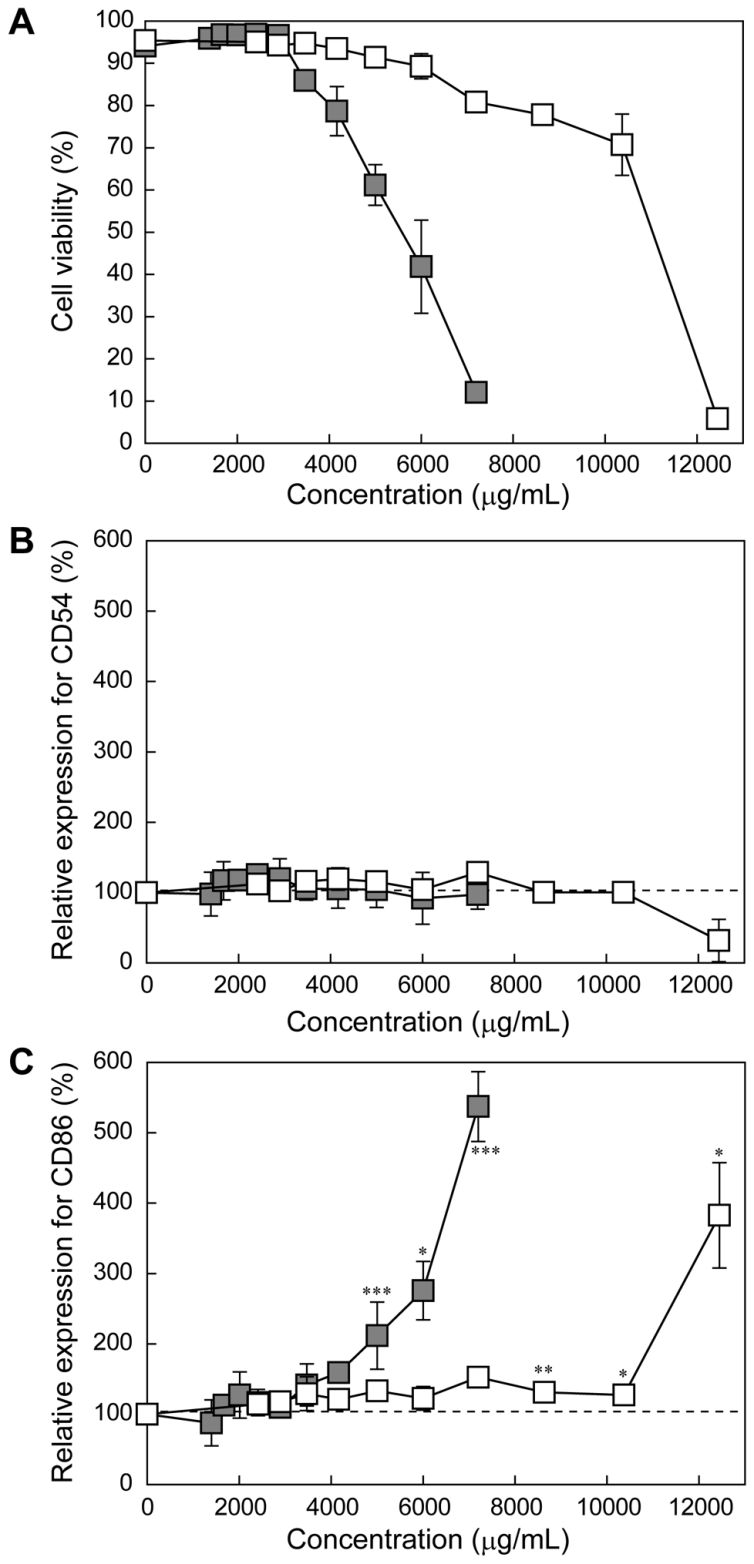
The effect of methacryloyl group of MMA on the expression of CD54 and CD86. Relative viability of (A) and expression levels of CD54 (B) and CD86 (C) in THP-1 cells treated with MMA (closed squares) or MIB (open squares) at various concentrations for 24 h. The plots for MMA were taken from Figure 2. The data are expressed as the means ± S.D. (n=3) (*p < 0.05, **p < 0.01, ***p < 0.005, ****p < 0.001).

It is known that dental resin monomers including triethylene glycol dimethacrylate (TEGDMA) and HEMA reduce the intracellular concentration of glutathione (GSH) and generate reactive oxygen species (ROS) in cells [25,26]. This monomer-induced oxidative stress is known to affect various cellular functions, such as cell cycle delay, induction of apoptosis, and activating the various cell signaling molecules, such as nuclear factor kappa B (NF-κB) [25]. To assess the effects of the double bonds in the methacryloyl groups of HEMA on the cell viability and the expression levels of CD54 and CD86, 2-hydroxyethyl isobutyrate (HEIB), an analog of HEMA possessing an isobutyrate moiety instead of a methacrylate moiety and thus lacking the double bond (Figure 1), was synthesized as a control. Note that the molecular weights of HEMA (MW: 130.14) and HEIB (MW: 132.16) are almost equal. The cytotoxicity measurements revealed that HEIB exhibited much lower cytotoxicity than HEMA (Figure 3A). HEIB induced significant expression of CD54 and CD86 at concentrations greater than 2,000 μg/mL (15.1 mM) and 1,500 μg/mL (11.3 mM), respectively, and the expression levels of both CD54 and CD86 increased in a concentration-dependent manner. The concentrations of HEIB that induced the significant expression of both CD54 and CD86 were higher than that of HEMA (Figure 3B, 3C), suggesting that HEIB has a lower immunostimulatory effect than HEMA. Notably, methyl isobutyrate (MIB), a non-double bond analog of MMA, showed tendencies similar to those of MMA with respect to cytotoxicity and the expression profiles of CD54 and CD86 (Figure 4). 

**Figure 5:**
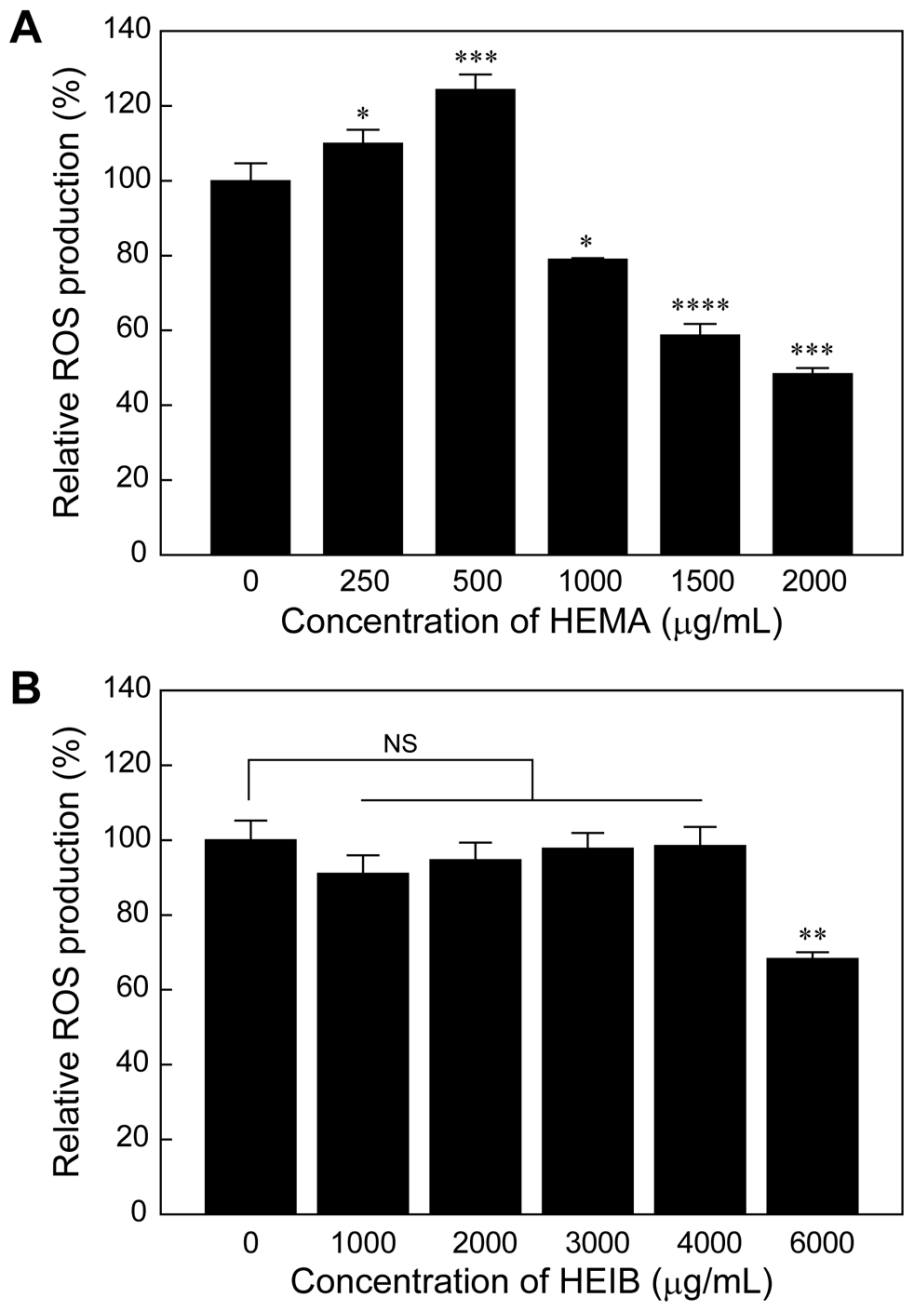
ROS production in THP-1 cells by the treatment with HEMA. Relative ROS levels in THP-1 cells treated with HEMA (A) or HEIB (B) at various concentrations for 24 h. The data are expressed as the means ± S.D. (n=3) (*p < 0.05, **p < 0.01, ***p < 0.005, ****p < 0.001).

 To assess the ROS generation in THP-1 cells due to exposure to HEMA or HEIB, the fluorescence intensity of 2’,7’-dichlorofluorescin diacetate (DCFH-DA) was determined after 24 h incubation with HEMA or HEIB. HEMA induced significant generation of ROS at 250 μg/mL (1.9 mM) and 500 μg/mL (3.8 mM) (Figure 5A). At concentrations greater than 1,000 μg/mL (7.6 mM), the fluorescence intensity of DCFH-DA decreased in a dose-dependent manner, presumably due to the effect of cell death. HEIB induced negligible generation of ROS at concentrations up to 4,000 μg/mL (30.3 mM). The fluorescence intensity of DCFH-DA decreased at concentrations greater than 6,000 μg/mL (45.4 mM), similar to the results for HEMA (Figure 5B).

### Expression of CD54 and CD86 induced by water-soluble PHEMA oligomers

**Figure 6:**
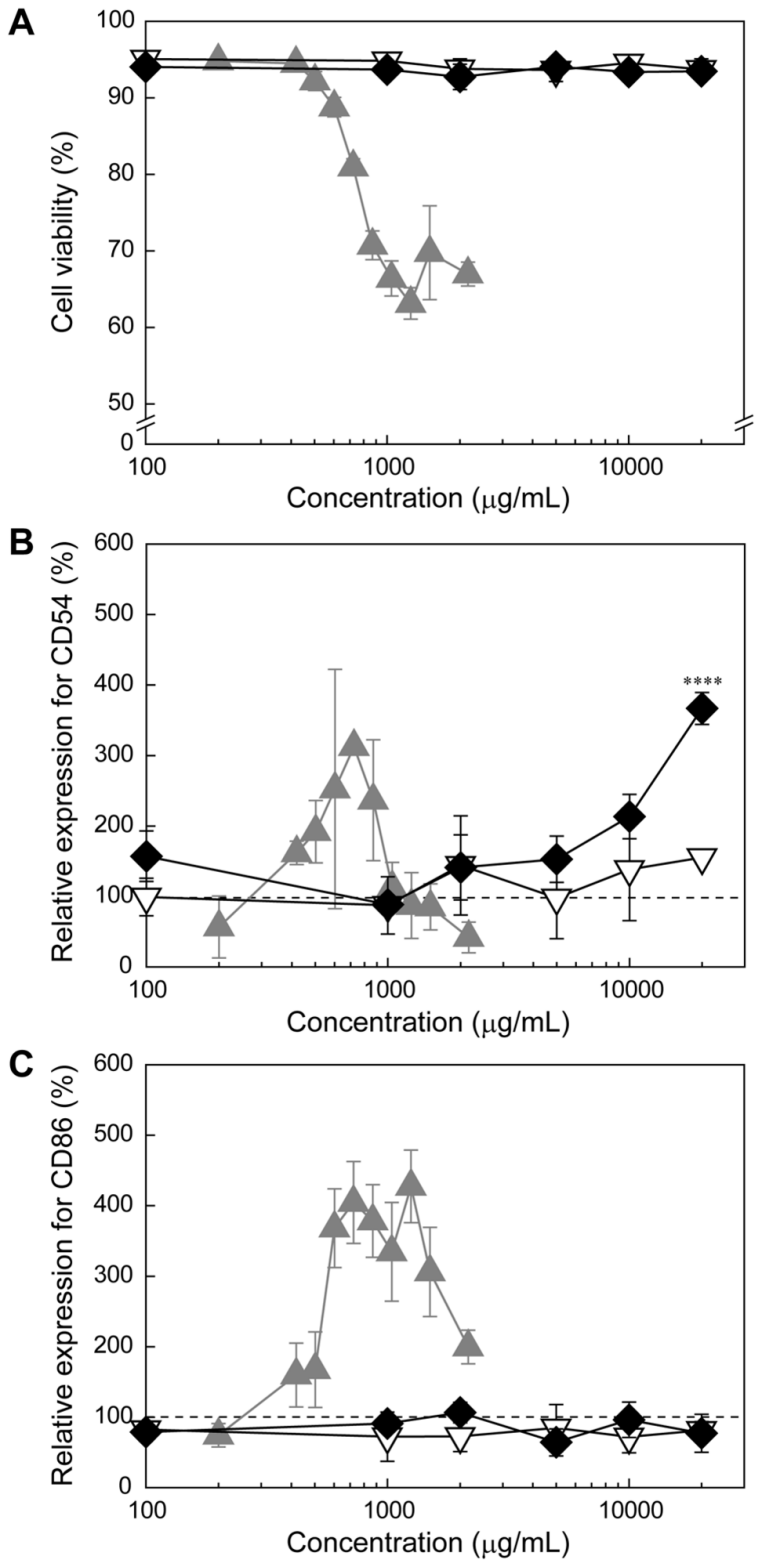
Expression of CD54 and CD86 in THP-1 cells by the treatment with water-soluble PHEMA oligomer. Relative viability of (A) and expression levels of CD54 (B) and CD86 (C) in THP-1 cells treated with HEMA (closed triangles), PHEMA15 (open triangles ), or PHEMA35 (closed diamonds) at various concentrations for 24 h. The plots for HEMA were taken from Figure 2. The data are expressed as the means ± S.D. (n=3) (*p < 0.05, **p < 0.01, ***p < 0.005, ****p < 0.001).

To evaluate whether the PHEMA has immunostimulatory effects on THP-1 cells, water-soluble PHEMA oligomers were synthesized by reversible addition-fragmentation chain transfer (RAFT) polymerization [27,28]. Two series of PHEMA were synthesized, and their degrees of polymerization, number-averaged molecular weights (*M*_n_), and polydispersity indices (PDI) are summarized in Table 1. Since the terminal dithiobenzoate groups were reported to show cytotoxicity [29], we completely converted the terminal dithiobenzoate to thiol groups, followed by the introduction of methyl groups. Although the dithiobenzoate-terminated PHEMA was not soluble in water, the substitution of the terminal dithiobenzoate group with a methyl group was found to provide the aqueous solubility to both PHEMA15 and PHEMA35. Accordingly, PHEMA15 and PHEMA35 exhibited negligible cytotoxicity due to dithiobenzoate, even at the concentration of 20,000 μg/mL (Figure 6A). PHEMA15 induced negligible expression of CD54 and CD86 at all tested concentrations. Additionally, PHEMA35 induced negligible expression of CD86 at all tested concentrations up to 20,000 μg/mL (Figure 6B, 6C). In contrast, PHEMA35 induced significant expression of CD54 at concentrations greater than 5,000 μg/mL, and the expression level increased in a dose-dependent manner. 

**Table 1:** Characterization of water-soluble PHEMA oligomers.

Code	Degree of polymerization**^a^**	*M*_n,NMR_^a^	*M*_w_/*M*_n_^b^
PHEMA15	15.3	2,220	1.24
PHEMA35	34.9	4,550	1.30

**^a^** Determined by ^1^H NMR in CD_3_OD. **^b^** Determined by SEC in DMSO.

## Discussion

In the previous reports, the effect of dental resin monomers on the viability of oral cells is investigated [5,6]. The purpose of this study is to estimate the sensitizing potentials of dental resin monomers, monomer derivatives, and polymers. In this regard, we investigated the effect of dental resin monomers, monomer derivatives, and polymers on the expression level of CD54 and CD86 in THP-1 cells, which is one of the in vitro assays to estimate the sensitization of chemical substrates [16–18]. NiSO_4_ is widely recognized as one of the most common contact allergens in humans [30], and there are many reports on allergic reactions caused by nickel ion [23,31,32]. Consistent with the previous reports, this study showed that nickel ion exhibited strong cytotoxicity and induced marked CD54 expression in THP-1 cells [18,21] (Figure 2). Additionally, NiSO_4_ was found to induce the expression of CD54 and CD86 at much lower concentrations than HEMA and MMA (Figure 2). This result indicates that the dental resin monomers have lower sensitizing potentials than NiSO_4_. These findings are consistent with the results of the clinical patch test study [4], in which the percentage of patients who were positive for nickel was higher than the percentages positive for resin-based materials. However, it is obvious that HEMA and MMA also induce the expression of CD54 or CD86, although higher concentrations are required that for NiSO_4_ (Figure 2). These results indicate that HEMA has a higher sensitization potential than MMA because HEMA induced the expression of both CD54 and CD86 at lower concentrations than MMA. This trend is consistent with the results of the clinical patch test study, in which the percentage of positive patch tests for HEMA was markedly higher than that for MMA [2,33]. It is hypothesized that the difference in the critical concentration required to induce the expression of CD54 and CD86 between HEMA and MMA is related to the monomer structure (i.e., methyl and hydroxyethyl groups).

It is known that the upregulation of CD54 is caused by the production of ROS [34], and the HEMA-induced expression of CD54 and CD86 is thought to be related to oxidative stress. Additionally, it has been revealed that HEMA increases the intracellular ROS level and depletes the intracellular GSH stores, leading to cell cycle perturbation and apoptosis [35–37]. The HEMA-induced cell cycle perturbation can be inhibited by the addition of *N*-acetyl-l-cysteine (NAC), in which the NAC undergoes Michael addition to methacryloyl group of HEMA and form NAC-HEMA adduct [38]. Herein, this cellular oxidative stress is also caused by Michael addition between a sulfhydryl group of GSH and the methacryloyl groups of the monomers [39]. To verify the effect of the double bond in the methacryloyl group on the immunostimulatory effect, a non-double bond-containing 2-hydroxyethyl isobutyrate (HEIB), was synthesized and its cytotoxicity, ROS production ability, and sensitizing potential were evaluated. HEMA induced ROS generation in THP-1 cells, which is consistent with the results of a previous study using human gingival epithelial S-G cells and pulp fibroblasts [35]. In contrast, HEIB induced negligible ROS production in comparison with HEMA because HEIB lacks the reactive methacryloyl group (Figure 5). Although HEIB induced some limited oxidative stress in THP-1 cells, HEIB was found to induce the expression of both CD54 and CD86 at higher concentrations than HEMA. The concentration dependence of HEIB on the expression levels of both CD54 and CD86 was similar to that of HEMA. Notably, the same tendency for the expression profiles of CD54 and CD86 was observed for MMA and MIB (Figure 4). These results indicate the HEIB- and MIB-induced expression of CD54 and CD86 might be related not only to oxidative stress but also to their inherent structure. Accordingly, the polymerized monomers may increase the expression level of CD54 even after their methacryloyl groups are no longer present.

Because the monomers exhibited higher cytotoxicity than the polymers, residual monomers may stimulate antigen-presenting cells to induce sensitization. Most dental resin-based polymers are not water soluble and are thought to show negligible erosion from the polymerized resin. However, in the case of PHEMA, the polymers are soluble in aqueous media when the degree of polymerization (or molecular weight) is low [40]. Thus, water-soluble PHEMA oligomers with different degrees of polymerization were used as a model to compare the sensitization potentials of monomer and polymer (Figure 6). Both PHEMA15 and PHEMA35 had negligible cytotoxicity in comparison with HEMA, confirming that the polymer is definitely not toxic. Additionally, PHEMA15 induced negligible expression of both CD54 and CD86, suggesting that PHEMA15 has no immunostimulatory effect. However, it is surprising that PHEMA35 induced significant expression of CD54 at high concentrations. Although the detailed mechanisms by which PHEMA35 induces the expression of CD54 are currently unclear, it may be speculated that high-molecular weight and water-soluble polymers are taken up to increase the intracellular concentration to stimulate some signaling pathway, resulting in the expression of CD54. This finding is considered important knowledge regarding the allergic responses to PHEMA oligomers.

In summary, our findings indicate that the representative dental resin monomers HEMA and MMA have lower sensitizing potentials than nickel ions. The comparison of HEMA and the non-double bond-containing compound HEIB suggested that the extent of sensitization to dental resin monomers estimated from CD54 and CD86 expression is related to the chemical structure of the monomer units, and the double bonds in the methacryloyl groups of the monomers strongly induced intracellular ROS production and cytotoxicity. Of special interest in this study is in the fact that water-soluble oligomers of HEMA induced CD54 expression at high concentrations despite the lack of cytotoxicity. These new findings provide insight into the nature of allergic responses to dental resin materials in clinical use and may facilitate the development of more biocompatible restorative materials in the future. 

## Materials and Methods

### Materials

2-Hydroxyethyl methacrylate (HEMA) was obtained from Kyoeisha Chemical (Osaka, Japan). Methyl methacrylate (MMA) was obtained from Wako Pure Chemical Industries (Osaka, Japan). Nickel(II) sulfate hexahydrate (NiSO_4_), 2,2'-azobis(isobutyronitrile) (AIBN), pyridine, ethylene glycol, and methyl iodide (MeI) were obtained from Kanto Chemicals (Tokyo, Japan). Methyl isobutyrate (MIB), isobutyryl chloride, and 2-hydroxyethylamine (HEA) were obtained from TCI (Tokyo Japan). 2-Cyano-2-propyl benzodithioate (CPB), 2’,7’-dichlorofluorescin diacetate (DCFH-DA), and propidium iodide (PI) were obtained from Aldrich (Milwaukee, WI, USA). The fluorescein isothiocyanate (FITC)-labeled mouse anti-human CD86 monoclonal antibody (clone: Fun-1) was obtained from BD Pharmingen (San Diego, CA, USA). The FITC-labeled mouse anti-human CD54 monoclonal antibody (clone: 6.5B5) and the FITC-labeled mouse IgG (clone: DAK-G01) were obtained from DAKO (Glostrup, Denmark). 

### Characterization of polymers

Size exclusion chromatography (SEC) was performed on an HLC-8120 system (Tosoh, Tokyo, Japan) equipped with a combination of TSKgel α-4000 and α-2500 columns (Tosoh). The eluent was dimethylsulfoxide (DMSO) containing 10 mM lithium bromide (LiBr) at a flow rate of 0.35 mL/min at 60 °C. The *M*_n,SEC_ and *M*_w_/*M*_n_ were calculated from a calibration curve of standard PEG samples (Agilent Technologies, Wilmington, DE, USA). ^1^H nuclear magnetic resonance (NMR) spectra were recorded on a Bruker Avance III 500 MHz spectrometer (Bruker BioSpin, Rheinstetten, Germany) in methanol-*d*_4_ (CD_3_OD) (Aldrich) at room temperature.

### Synthesis of 2-hydroxyethyl isobutyrate (HEIB)

Ethylene glycol (118 g, 1.90 mol) and pyridine (7.52 g, 95.1 mmol) were loaded into a flask under a nitrogen atmosphere. Isobutyryl chloride (10.1 g, 95.1 mmol) was added dropwise to this mixture in an ice bath, and the system was stirred for 3 h at room temperature [41]. After the reaction, water (400 mL) was added to the reaction mixture, and the product was extracted three times with diethyl ether (200 mL). The organic layer was concentrated and dried over MgSO_4_. Then, the product was distilled under reduced pressure to obtain the purified product as a colorless oil (5.21 g, 41.5%). ^1^H NMR (500 MHz, CD_3_OD): δ = 1.04 (6H, d, *J* = 6.9 Hz, -CH(C***H*_*3*_**)_2_), 2.49 (1H, m, -C***H***(CH _3_)_2_), 3.51 (2H, q, *J* = 5.2 Hz, -O-CH_2_-C***H*_*2*_**-OH), 3.97 (2H, t, *J* = 5.2 Hz, -O-C***H*_*2*_**-CH_2_-OH), 4.74 (1H, t, *J* = 5.5 Hz, -O***H***).

### Synthesis of water-soluble poly (2-hydroxyethyl methacrylate) oligomers

Water-soluble PHEMA oligomers were synthesized by reversible addition-fragmentation chain transfer (RAFT) polymerization [27,28]. Immediately before polymerization, the HEMA was purified by passing through an inhibitor removal column (Aldrich). Then, HEMA (3.00 g, 23.1 mmol for both PHEMA15 and PHEMA35), AIBN (87.5 mg, 688 μmol for PHEMA15 and 19.5 mg, 154 μmol for PHEMA35), and CPB (762 mg, 3.44 mmol for PHEMA15 and 170 mg, 768 μmol for PHEMA35) were dissolved in ethanol (11.5 mL for PHEMA15 and PHEMA35), and the solution was deoxygenated by subjecting it to three freeze-thaw cycles. The reaction mixture was stirred for 18 h at 70 °C. After polymerization, the polymer was purified by reprecipitation in diethyl ether. The recovered polymers were dried under reduced pressure to obtain PHEMA as a powder (1.06 g for PHEMA15 and 1.58 g for PHEMA35). The *M*_n,SEC_ and *M*_w_/*M*_n_ of the polymers were determined by SEC. The *M*_n,NMR_ and degree of polymerization (DP) were determined from the ^1^H NMR spectra. ^1^H NMR (500 MHz, CD_3_OD): δ = 0.69-1.46 (m, -C(CH_*3*_)-), 1.46-2.28 (m, -C***H*_*2*_**-C(CH_3_)-), 3.55-3.90 (m, -O-CH_2_-C***H*_*2*_**-OH), 3.90-4.34 (m, -O-C***H*_*2*_**-CH_2_-OH), 7.43 (m, dithiobenzoate), 7.60 (m, dithiobenzoate, 7.89 (m, dithiobenzoate).

Then, the polymers (0.5 g, 225 μmol for PHEMA15 and 1.0 g, 220 μmol for PHEMA35) were dissolved in N,N-dimethylformamide (10 mL), and HEA (136 μL, 2.25 mmol) was added to the solution. After 2 h of stirring, MeI (701 μL, 11.3 μmol for PHEMA15 and 684 μL, 11.0 mmol for PHEMA35) was added to the solution, which was then stirred overnight at room temperature. Then, the reaction mixture was dialyzed against pure water for 3 days to remove unreacted reagents (Spectra/Por Biotech, molecular weight cut-off of 500) (Spectrum Laboratories, Rancho Dominguez, CA). Finally, the aqueous solution was lyophilized from water to obtain the water-soluble PHEMA as a white powder (425 mg for PHEMA15 and 949 mg for PHEMA35). The chemical composition was determined from the integral ratios for the ^1^H NMR spectra. ^1^H NMR (500 MHz, CD_3_OD): δ = 0.69-1.46 (m, -C(CH_*3*_)-), 1.46-2.28 (m, -C***H*_*2*_**-C(CH_3_)-), 3.55-3.90 (m, -O-CH_2_-C***H*_*2*_**-OH), 3.90-4.34 (m, -O-C***H*_*2*_**-CH_2_-OH).

### Cell culture

THP-1 cells, derived from a human monocytic leukemia, were obtained from the American Type Culture Collection (Manassas, VA, USA) and grown in RPMI 1640 medium (Gibco BRL, Grand Island, NY, USA) containing 10% heat-inactivated fetal bovine serum (FBS) (Gibco BRL), 100 units/mL penicillin, 100 µg/mL streptomycin (Gibco BRL), and 50 μM 2-mercaptoethanol (Wako) in a humidified 5% CO_2_ atmosphere at 37 °C. The cells were pre-cultured in non-adhesive culture bottles (Sumitomo Bakelite, Tokyo, Japan) at a cell density range of 1×10^5^ to 2×10^5^ cells/mL. 

### Flow cytometry

 THP-1 cells were seeded in a 24-well non-adhesive culture plate (Becton Dickinson, Franklin Lakes, NJ, USA) at a density of 1.0×10^6^ cells/well, followed by the addition of test solutions to each well. After 24 h of incubation, the cells were washed twice with phosphate-buffered saline (PBS) containing 0.1% bovine serum albumin (BSA). The cells were treated with 0.01% globulins (Cohn fraction II, III) (Sigma-Aldrich, St. Louis, MO, USA) for 15 min on ice to block the Fc receptor. The cell suspension was divided into three portions, which were stained with FITC-labeled CD54, CD86, and IgG antibodies, respectively, for 30 min on ice. Then, the cells were washed twice with PBS containing 0.1% BSA and stained with PI (0.625 µg/mL) for gating out dead cells as well as determining cell viability. Flow cytometric analysis was performed on a FACSCanto II (Becton Dickinson) to determine the cell viability and mean fluorescence intensity (MFI) of each group of cells. The relative fluorescence intensity (RFI) of the cells was calculated as follows: RFI = ([MFI_sample_ of CD54 or CD86] – [MFI_sample_ of IgG])/([MFI_control_ of CD54 or CD86] – [MFI_control_ of IgG]) × 100, where MFI_sample_ and MFI_control_ represent the MFI of sample-treated and non-treated cells, respectively. Each test was performed in triplicate, and 10,000 living cells were analyzed to determine the MFI of the cell populations.

### Reactive oxygen species (ROS) measurements

 THP-1 cells were seeded in 24-well non-adhesive culture plate at a density of 0.3×10^6^ cells/well, and then, test solutions were added to each well. After 24 h of incubation, the cells were washed with PBS containing 0.1% BSA, followed by treatment with 20 μM DCFH-DA for 30 min at 37 °C. After incubation, the cells were washed with PBS containing 0.1% BSA. The MFI of DCFH-DA in cells was determined by flow cytometry on a FACSCanto II. The ROS generation was calculated relative to that of untreated cells. Each test was performed in triplicate.

### Statistical analysis

Statistical analysis was performed using a two-tail Student’s *t*-test. A *p*-value of less than 0.05 was considered indicative of statistical significance. The values are expressed as the mean ± standard deviation (S.D.).
